# Hormonal Activity of 3D Bioprinting Materials Using the E-Screen and PALM Bioassays

**DOI:** 10.3390/toxics14090760

**Published:** 2026-08-26

**Authors:** Alicia Olivas-Martinez, Elisa Nygren-Jiménez, Jose Manuel Molina-Molina, Gema Jiménez, Mariana F. Fernández, Juan Antonio Marchal

**Affiliations:** 1 Instituto de Investigación Biosanitaria de Granada (ibs.GRANADA), 18012 Granada, Spain; aolivas@ugr.es (A.O.-M.); elisanygren@go.ugr.es (E.N.-J.); molinajm@ugr.es (J.M.M.-M.); gemajg@ugr.es (G.J.); jmarchal@go.ugr.es (J.A.M.); 2 Centro de Investigación Biomédica en Red de Epidemiología y Salud Pública (CIBERESP), 28029 Madrid, Spain; 3 Department of Radiology and Physical Medicine, School of Medicine, University of Granada, 18016 Granada, Spain; 4 Biopathology and Regenerative Medicine Institute (IBIMER), University of Granada, 18100 Granada, Spain; 5 Department of Human Anatomy and Embryology, School of Medicine, University of Granada, 18016 Granada, Spain; 6 BioFab i3D Lab—Biofabrication and 3D (Bio)printing Laboratory, 18016 Granada, Spain; 7 Excellence Research Unit “Modeling Nature” (MNat), University of Granada, 18016 Granada, Spain

**Keywords:** hormonal activity, endocrine disruption, E-screen assay, 3D printing, bioprinting, biomaterials

## Abstract

Three-dimensional (3D) printing and bioprinting technologies are increasingly used in tissue engineering and in the development of personalized medical devices with tailored biological and mechanical properties. Despite their advantages, these technologies may involve exposure to chemical compounds with uncertain biological effects, including potential endocrine-disrupting activity. This study aims to evaluate the hormonal activity and cytotoxicity of various materials commonly used for 3D printing technologies, including thermoplastics and synthetic materials (PLA, ABS, PCL, b-TPUe, PVA, PEGDA), and biomaterials (collagen, alginate, hyaluronic acid, agarose, silk fibroin, GelMA, HAMA). Hormonal activity was evaluated using the E-screen assay for estrogenic and anti-estrogenic activity, and the PALM assay for androgenic and anti-androgenic activity. Cytotoxic effects are evaluated by assessing cellular metabolic viability using the MTT assay. No detectable estrogenic, anti-estrogenic, androgenic, or anti-androgenic activity, nor any signs of cytotoxicity, were observed within the experimental conditions analysed. This study represents the first analysis of some specific endocrine-related endpoints of a wide range of 3D printing and bioprinting materials, and should not be interpreted as definitive proof of the complete absence of endocrine activity. Further studies addressing additional toxicological parameters and long-term assessments are required to comprehensively characterize their safety and suitability.

## 1. Introduction

Three-dimensional (3D) printing and bioprinting are transforming current clinical practice by enabling tissue fabrication and the generation of 3D structures with biological and mechanical properties suitable for restoring tissue and organ function. In addition, these technologies facilitate the design of personalized medical devices generated from the patient’s own cells, allowing the creation of personalized functional constructs [1]. 3D printing enables the layer-by-layer fabrication of structures, while bioprinting applies this approach to bioinks formulated with natural and/or synthetic materials that can encapsulate cells, enabling the generation of constructs with tailored biological and mechanical properties [2,3].

The use of these emerging technologies may involve exposure to chemical compounds with potentially uncharacterized biological effects. For example, thin polycarbonate splints, which are characterized by high strength, hardness, and durability, may release relevant amounts of bisphenol A (BPA), the main component of the polymer and a well-known endocrine disruptor [4,5]. Exposure to BPA and other endocrine-disrupting chemicals from medical devices has been previously reported [4,6].

The presence of endocrine disruptors in these new materials may therefore pose potential health risks, especially if exposure occurs during critical developmental stages or is prolonged over time. Such exposure may contribute to diseases linked to disruptions in hormonal homeostasis [7,8]. To address this concern, we evaluated the hormonal activity and cytotoxicity of thirteen natural and synthetic materials commonly employed in the generation of bioprinted tissues, organs and medical devices [3].

## 2. Materials and Methods

The materials analyzed included polylactic acid (PLA) [9,10], acrylonitrile butadiene styrene (ABS) [10], polycaprolactone (PCL) [11], 1,4-butanediol thermoplastic polyurethane elastomer (b-TPUe) [12,13], polyvinyl alcohol (PVA) [14], poly(ethylene glycol) diacrylate (PEGDA) [15], collagen [16,17], alginate [14,16,18], hyaluronic acid [19], agarose [17], silk fibroin [18], methacrylated gelatine (GelMA) [15,20] and methacrylated hyaluronic acid (HAMA) [20,21]. Briefly, the tested materials were dissolved according to their solubility properties. PLA, ABS, PCL, and b-TPUe were dissolved in dimethyl sulfoxide (DMSO); PEGDA, collagen, alginate, hyaluronic acid, agarose, silk fibroin, GelMA, and HAMA were dissolved in phosphate-buffered saline (PBS); and PVA was dissolved in double-distilled water (DDW). When required, specific dissolution conditions were applied (e.g., incubation at 4 °C, 37 °C or 60 °C, heating, and/or sonication).

Hormonal activities were evaluated through the E-screen and PALM assays as described by Molina-Molina et al. [22,23]. Briefly, estrogenic and anti-estrogenic activities (E-Screen) were assessed in the human breast cancer cell line MCF-7, a well-established estrogen-responsive model, by measuring cell proliferation in the presence or absence of 17β-estradiol. Anti-estrogenic activity was determined by the ability of the compounds to inhibit estradiol-induced proliferation. Androgenic and anti-androgenic activities were evaluated using the androgen-dependent PALM cells by measuring changes in luciferase activity following exposure to the test compounds. Anti-androgenic effects were determined by coincubation with the reference androgen metiltrienolone (R1881). Additionally, cellular metabolic viability following exposure to the tested compounds was assessed using the MTT assay, based on the modified method of Denizot and Lang [24]. Stock solutions of the tested materials were subsequently serially diluted in the corresponding test culture medium before biological testing. For all experimental conditions, including the test materials and positive controls, the final solvent concentration in the culture medium did not exceed 0.1% (*v*/*v*). Moreover, each treatment was compared with its corresponding vehicle control, ensuring that any observed biological effects were attributable to the tested materials rather than to the solvent. Maximum concentrations of the tested materials were within or slightly below those reported for bioinks in the literature [14,15,16,17,18,19,20,21], while thermoplastic polymers were tested at a molar concentration equal to the maximum used for the biopolymers. This approach enabled testing under relevant conditions for bioprinting applications while maintaining fully dissolved solutions.

All experiments were performed in three independent experiments, with triplicate (E-screen and MTT) or quadruplicate (PALM) measurements for each concentration. Data are presented as mean ± standard deviation (SD). Statistical significance was assessed using one-way ANOVA followed by Dunnett’s post hoc test, with *p* < 0.05 considered statistically significant.

## 3. Results and Discussion

The materials analyzed included synthetic polymers: PLA [9,10], ABS [10], PCL [11], b-TPUe [12,13], PVA [14] and PEGDA [15], as well as natural biomaterials, including chemically modified biomaterials commonly used in bioinks: collagen [16,17], alginate [14,16,18], hyaluronic acid [19], agarose [17], silk fibroin [18], GelMA [15,20] and HAMA [20,21].

The effects of the evaluated materials in the E-screen assay are summarized in Table 1. Tested concentrations were selected according to the solubility of each material and reported concentrations for bioinks in the literature [14,15,16,17,18,19,20,21]. Across all tested concentrations, none of the materials showed detectable estrogenic activity comparable to that of the positive control, with values remaining close to the baseline, even at the higher concentrations tested. When the materials were grouped by type, no clear trend towards increased activity at higher concentrations was observed when synthetic materials were compared with natural ones. For anti-estrogenic activity, all materials yielded values comparable to the positive control, with no concentration-dependent decrease in proliferation observed, and no material types showing a tendency toward anti-estrogenic behaviour. The results in the PALM assay showed similar results (Table 2), with all tested materials yielding androgenic activity values close to the negative controls. When the compounds were tested for their ability to antagonize human androgen receptor (hAR) in our cell model, no antagonistic activity was detected. None of the tested materials produced a meaningful dose-dependent change in activity, nor was any clear difference observed between synthetic and natural materials. All positive and negative controls yielded the expected results, confirming the validity of the assays.

Furthermore, MTT assays showed no significant effects on the metabolic viability of MCF-7 and PALM cells, with viability values ranging from 95% to 100% at all tested concentrations, confirming the absence of relevant cytotoxic effects (Supplementary Tables S1 and S2).

Our results indicate that, within the tested concentration ranges and experimental conditions analyzed, no detectable estrogenic, anti-estrogenic, androgenic, or anti-androgenic activity, nor significant toxicity, was observed for all the biomaterials tested. The concentrations evaluated in this study should be interpreted as screening concentrations under controlled conditions and do not necessarily reflect in vivo exposure levels. Further studies evaluating compound release from printed constructs and their long-term biological effects are required to determine the relevance of these findings under realistic exposure scenarios.

## 4. Conclusions

This study represents the first in vitro screening assessment of selected endocrine activities—specifically, estrogenic and anti-androgenic activities—in a wide range of materials used in 3D printing and bioprinting. The aim of this work was to provide an initial assessment, rather than a comprehensive evaluation of the endocrine-disrupting potential or overall biological safety of these materials. Under the experimental conditions and concentration ranges tested, no detectable estrogenic, anti-estrogenic, androgenic or anti-androgenic activity was observed through the estrogen and androgen receptor pathways. However, these findings should not be interpreted as definitive evidence of the complete absence of endocrine activity. Further studies addressing additional toxicological and biological parameters, as well as long-term in vitro and in vivo assessments, are required to comprehensively characterize their safety and suitability for 3D-printing and bioprinting applications.

## Figures and Tables

**Table 1:** Proliferative and anti-proliferative effects of 13 bioprinting materials measured by the E-screen assay in MCF-7 cells.

		PLA	ABS	PCL	b-TPUe	PEDGA	Hyaluronic Acid	Agarose	Silk Fibroin	GelMa	HAMA	PVA	Collagen	Alginate
Estrogenic activity	C+	6.36 * ± 0.06	6.46 * ± 0.34	6.45 * ± 0.22	6.53 * ± 0.21	6.36 * ± 0.17	6.51 * ± 0.04	6.39 * ± 0.36	6.53 * ± 0.16	6.24 * ± 0.11	6.17 * ± 0.11	6.51 * ± 0.04	6.76 * ± 0.10	6.51 * ± 0.21
	C−	1.00 ± 0.04	1.00 ± 0.11	1.00 ± 0.06	1.00 ± 0.03	1.00 ± 0.04	1.00 ± 0.01	1.00 ± 0.01	1.00 ± 0.01	1.00 ± 0.02	1.00 ± 0.04	1.00 ± 0.03	1.00 ± 0.10	1.00 ± 0.01
	10^−9^ M	N.T.										1.04 ± 0.02	1.03 ± 0.03	1.09 ± 0.05
	3 × 10^−9^ M	N.T.										1.10 ± 0.03	1.09 ± 0.03	1.11 ± 0.06
	10^−8^ M	1.02 ± 0.03	1.05 ± 0.05	1.03 ± 0.04	1.04 ± 0.01	1.08 ± 0.01	1.02 ± 0.02	1.06 ± 0.01	1.04 ± 0.02	1.06 ± 0.01	1.01 ± 0.01	1.07 ± 0.03	1.13 ± 0.04	1.11 ± 0.01
	3 × 10^−8^ M	1.04 ± 0.03	1.05 ± 0.06	1.04 ± 0.04	1.09 ± 0.01	1.09 ± 0.02	1.07 ± 0.02	1.07 ± 0.01	1.06 ± 0.03	1.12 ± 0.03	1.05 ± 0.01	1.11 ± 0.01	1.06 ± 0.02	1.10 ± 0.01
	10^−7^ M	1.08 ± 0.04	1.11 ± 0.04	1.05 ± 0.01	1.08 ± 0.01	1.08 ± 0.04	1.08 ± 0.06	1.06 ± 0.01	1.11 ± 0.01	1.08 ± 0.03	1.06 ± 0.07	1.06 ± 0.01	1.08 ± 0.06	1.07 ± 0.01
	3 × 10^−7^ M	1.09 ± 0.04	1.07 ± 0.06	1.08 ± 0.03	1.09 ± 0.03	1.10 ± 0.01	1.04 ± 0.04	1.08 ± 0.05	1.07 ± 0.01	1.11 ± 0.01	1.05 ± 0.03	1.10 ± 0.01	1.09 ± 0.01	1.09 ± 0.03
	10^−6^ M	1.08 ± 0.01	1.04 ± 0.04	1.05 ± 0.02	1.10 ± 0.01	1.12 ± 0.05	1.11 ± 0.04	1.10 ± 0.04	1.14 ± 0.04	1.04 ± 0.04	1.09 ± 0.08	1.08 ± 0.06	1.09 ± 0.01	1.12 ± 0.01
	3 × 10^−6^ M	1.05 ± 0.01	1.12 ± 0.02	1.08 ± 0.02	1.06 ± 0.01	1.07 ± 0.04	1.06 ± 0.04	1.11 ± 0.01	1.08 ± 0.07	1.13 ± 0.02	1.10 ± 0.06	N.T.		
	10^−5^ M	1.07 ± 0.01	1.06 ± 0.03	1.09 ± 0.02	1.10 ± 0.01	1.10 ± 0.01	1.07 ± 0.02	1.09 ± 0.04	1.14 ± 0.05	1.10 ± 0.05	1.09 ± 0.02	N.T.		
Anti-estrogenic activity^+^	C+	6.49 ± 0.44	6.53 ± 0.24	6.47 ± 0.10	6.46 ± 0.12	6.51 ± 0.03	6.44 ± 0.02	6.59 ± 0.02	6.58 ± 0.03	6.53 ± 0.21	6.60 ± 0.11	6.62 ± 0.37	6.48 ± 0.13	6.61 ± 0.13
	C−	1.00 * ± 0.01	1.00 * ± 0.03	1.00 * ± 0.10	1.00 * ± 0.01	1.00 * ± 0.02	1.00 * ± 0.01	1.00 * ± 0.11	1.00 * ± 0.01	1.00 * ± 0.03	1.00 * ± 0.04	1.00 * ± 0.01	1.00 * ± 0.02	1.00 * ± 0.01
	10^−9^ M	N.T.										6.70 ± 0.09	6.48 ± 0.26	6.60 ± 0.11
	3 × 10^−9^ M	N.T.										6.55 ± 0.12	6.57 ± 0.06	6.73 ± 0.24
	10^−8^ M	6.72 ± 0.03	6.58 ± 0.35	6.43 ± 0.21	6.58 ± 0.01	6.44 ± 0.21	6.36 ± 0.01	6.61 ± 0.18	6.62 ± 0.37	6.37 ± 0.25	6.50 ± 0.08	6.64 ± 0.10	6.71 ± 0.06	6.50 ± 0.26
	3 × 10^−8^ M	6.47 ± 0.01	6.74 ± 0.20	6.57 ± 0.02	6.53 ± 0.01	6.53 ± 0.21	6.50 ± 0.04	6.45 ± 0.02	6.76 ± 0.20	6.62 ± 0.26	6.57 ± 0.13	6.35 ± 0.03	6.32 ± 0.12	6.58 ± 0.19
	10^−7^ M	6.59 ± 0.06	6.75 ± 0.11	6.53 ± 0.04	6.53 ± 0.13	6.49 ± 0.37	6.56 ± 0.03	6.69 ± 0.18	6.61 ± 0.13	6.67 ± 0.24	6.51 ± 0.08	6.77 ± 0.11	6.57 ± 0.04	6.53 ± 0.24
	3 × 10^−7^ M	6.66 ± 0.05	6.77 ± 0.14	6.54 ± 0.23	6.61 ± 0.08	6.43 ± 0.16	6.45 ± 0.05	6.63 ± 0.16	6.68 ± 0.28	6.59 ± 0.12	6.37 ± 0.21	6.65 ± 0.17	6.77 ± 0.02	6.54 ± 0.25
	10^−6^ M	6.48 ± 0.09	6.59 ± 0.25	6.54 ± 0.33	6.54 ± 0.33	6.50 ± 0.37	6.63 ± 0.03	6.60 ± 0.09	6.80 ± 0.17	6.53 ± 0.17	6.63 ± 0.21	6.65 ± 0.12	6.52 ± 0.04	6.54 ± 0.06
	3 × 10^−6^ M	6.58 ± 0.09	6.72 ± 0.46	6.59 ± 0.12	6.59 ± 0.07	6.42 ± 0.17	6.58 ± 0.05	6.56 ± 0.01	6.54 ± 0.20	6.43 ± 0.18	6.45 ± 0.01	N.T.		
	10^−5^ M	6.49 ± 0.01	6.60 ± 0.04	6.66 ± 0.22	6.49 ± 0.05	6.57 ± 0.12	6.62 ± 0.14	6.59 ± 0.09	6.60 ± 0.12	6.58 ± 0.39	6.64 ± 0.14	N.T.		

C+: positive control (E2 1 × 10^−10^ M); C−: negative control (culture media); N.T.: not tested; SD: Standard deviation. *: Significant difference compared to C− (estrogenic activity) or C+ (anti-estrogenic activity) (*p* < 0.05). Results are expressed as MCF-7 cell proliferation (fold over control), calculated as the ratio between the maximum proliferation induced by the sample and that of the negative control (C−). +For anti-estrogenic activity, MCF-7 cells were treated with the bioprinting materials and E2 (100 pM). ABS: acrylonitrile butadiene styrene; b-TPUe: 1,4-butanediol thermoplastic polyurethane elastomer; GelMA: methacrylated gelatine; HAMA: methacrylated hyaluronic acid; PCL: polycaprolactone; PEGDA: poly(ethylene glycol) diacrylate; PLA: polylactic acid; PVA: polyvinyl alcohol.

**Table 2:** Transcriptional activity of the human androgen receptor (hAR) of 13 bioprinting materials measured by the PALM assay.

		PLA	ABS	PCL	b-TPUe	PEDGA	Hyaluronic Acid	Agarose	Silk Fibroin	GelMa	HAMA	PVA	Collagen	Alginate
Androgenic activity	C+	106.59 * ± 5.37	95.40 * ± 0.28	100.83 * ± 0.56	100.27 * ± 0.95	99.46 * ± 0.94	101.29 * ± 4.27	101.61 * ± 1.21	99.62 * ± 5.83	98.64 * ± 3.55	96.18 * ± 7.00	99.78 * ± 0.81	99.43 * ± 0.93	102.59 * ± 4.31
	C−	10.21 ± 0.24	9.68 ± 0.11	9.81 ± 0.69	10.05 ± 0.07	10.56 ± 1.34	10.04 ± 0.65	10.00 ± 1.50	9.24 ± 1.13	9.81 ± 0.36	9.31 ± 0.30	10.64 ± 0.93	9.64 ± 0.87	9.78 ± 0.21
	10^−9^ M	N.T.										10.63 ± 0.67	9.96 ± 0.28	10.16 ± 0.32
	3 × 10^−9^ M	N.T.										10.85 ± 0.76	10.16 ± 0.32	9.45 ± 0.13
	10^−8^ M	9.82 ± 1.46	9.06 ± 0.16	10.20 ± 0.72	10.40 ± 0.43	10.46 ± 0.77	9.86 ± 0.73	10.02 ± 1.35	9.16 ± 1.12	10.03 ± 0.28	9.35 ± 0.13	10.41 ± 0.77	10.60 ± 0.50	10.30 ± 0.86
	3 × 10^−8^ M	9.63 ± 0.48	9.77 ± 0.34	10.38 ± 0.48	10.23 ± 0.38	10.84 ± 1.12	10.21 ± 0.76	10.16 ± 1.42	9.58 ± 0.84	9.90 ± 0.08	9.74 ± 0.22	10.47 ± 0.84	10.16 ± 0.51	9.24 ± 0.69
	10^−7^ M	10.73 ± 0.95	9.63 ± 0.13	10.31 ± 0.28	10.39 ± 0.45	10.49 ± 1.24	10.44 ± 1.04	9.94 ± 1.11	9.86 ± 1.23	9.93 ± 0.56	9.88 ± 0.37	10.17 ± 1.47	10.22 ± 0.84	9.86 ± 0.32
	3 × 10^−7^ M	10.11 ± 0.06	9.61 ± 0.92	10.08 ± 0.24	10.04 ± 0.08	10.66 ± 0.09	10.07 ± 0.71	9.86 ± 1.38	9.57 ± 0.84	10.23 ± 0.42	10.04 ± 0.38	10.35 ± 1.44	10.0 ± 0.67	9.83 ± 0.68
	10^−6^ M	9.68 ± 1.22	9.66 ± 1.24	10.42 ± 0.89	9.90 ± 0.85	10.53 ± 1.29	10.11 ± 1.32	10.21 ± 0.59	9.76 ± 1.17	10.49 ± 0.80	9.46 ± 0.87	10.95 ± 1.67	10.93 ± 0.63	10.09 ± 0.99
	3 × 10^−6^ M	10.33 ± 0.63	9.44 ± 0.02	10.13 ± 0.32	10.29 ± 0.43	10.40 ± 1.50	10.16 ± 0.83	10.47 ± 0.93	9.84 ± 0.58	9.86 ± 0.85	10.06 ± 0.81	N.T.		
	10^−5^ M	11.14 ± 0.20	9.78 ± 0.65	10.05 ± 1.34	9.81 ± 0.27	10.29 ± 1.29	10.66 ± 1.76	11.23 ± 0.93	10.31 ± 1.41	10.61 ± 1.86	9.95 ± 0.87	N.T.		
Anti-androgenic activity^+^	C+	81.79 ± 0.10	82.81 ± 0.45	81.82 ± 1.49	83.28 ± 1.46	88.14 ± 0.03	82.40 ± 0.89	80.34 ± 1.47	81.08 ± 1.50	80.91 ± 0.22	81.03 ± 0.05	78.79 ± 1.91	80.80 ± 0.80	81.18 ± 0.32
	C−	10.05 * ± 0.20	9.95 * ± 0.23	10.20 * ± 0.10	10.11 * ± 0.14	10.13 * ± 0.12	10.11 * ± 0.21	9.89 * ± 0.11	10.25 * ± 0.25	9.88 * ± 0.13	10.02 * ± 0.08	10.23 * ± 0.21	10.33 * ± 0.12	9.98 * ± 0.25
	10^−9^ M	N.T.										80.92 ± 0.10	80.88 ± 0.84	81.54 ± 1.02
	3 × 10^−9^ M	N.T.										79.15 ± 1.12	82.24 ± 1.51	81.80 ± 0.43
	10^−8^ M	83.72 ± 0.10	81.10 ± 0.45	83.02 ± 2.78	80.98 ± 1.33	82.28 ± 2.04	80.42 ± 0.84	81.66 ± 0.36	81.08 ± 0.97	82.45 ± 0.32	81.20 ± 0.48	81.40 ± 1.02	80.22 ± 1.86	80.17 ± 1.82
	3 × 10^−8^ M	83.77 ± 0.47	83.03 ± 0.79	83.63 ± 1.39	84.56 ± 0.69	82.07 ± 0.52	82.73 ± 1.58	82.74 ± 0.34	83.40 ± 1.46	83.00 ± 1.84	84.21 ± 2.17	81.55 ± 0.84	81.17 ± 2.97	82.29 ± 1.72
	10^−7^ M	81.57 ± 0.34	80.79 ± 1.34	83.71 ± 1.75	82.92 ± 0.03	82.99 ± 2.45	81.97 ± 1.01	83.49 ± 2.60	81.82 ± 0.28	82.38 ± 0.60	82.61 ± 0.96	80.90 ± 3.33	82.81 ± 2.24	79.99 ± 0.42
	3 × 10^−7^ M	85.02 ± 0.48	82.35 ± 1.17	81.66 ± 1.75	82.59 ± 2.50	80.94 ± 1.03	81.80 ± 1.68	81.42 ± 0.91	81.76 ± 0.82	82.20 ± 0.24	82.11 ± 0.75	80.10 ± 2.49	82.06 ± 1.34	83.00 ± 0.44
	10^−6^ M	83.54 ± 0.47	84.20 ± 0.90	83.55 ± 1.18	84.33 ± 2.15	82.82 ± 0.45	81.56 ± 1.46	83.50 ± 0.59	82.23 ± 1.34	82.76 ± 0.19	82.44 ± 2.40	81.80 ± 0.35	80.56 ± 0.83	81.51 ± 2.50
	3 × 10^−6^ M	81.55 ± 0.46	80.76 ± 0.45	83.69 ± 1.86	82.89 ± 1.83	82.97 ± 2.57	81.72 ± 3.18	83.90 ± 2.81	82.38 ± 3.08	82.96 ± 1.05	82.06 ± 2.20	N.T.		
	10^−5^ M	82.92 ± 0.46	80.65 ± 1.47	82.18 ± 2.38	82.76 ± 0.13	81.53 ± 1.76	82.27 ± 0.25	82.19 ± 1.90	81.68 ± 1.90	82.98 ± 1.23	81.51 ± 2.58	N.T.		

C+: positive control (R1881 1 × 10^−7^ M); C−: negative control (culture media); N.T.: not tested; SD: Standard deviation. *: Significant difference compared to C− (androgenic activity) or C+ (anti-androgenic activity) (*p* < 0.05). Results are expressed as maximal luciferase activity (%), where 100% corresponds to the luciferase activity induced by 100 nM R1881. + For anti-androgenic activity, PALM cells were treated with the bioprinting materials and R1881 (0.3 nM). ABS: acrylonitrile butadiene styrene; b-TPUe: 1,4-butanediol thermoplastic polyurethane elastomer; GelMA: methacrylated gelatine; HAMA: methacrylated hyaluronic acid; PCL: polycaprolactone; PEGDA: poly(ethylene glycol) diacrylate; PLA: polylactic acid; PVA: polyvinyl alcohol.

## Data Availability

The data that support the findings of this study are available from the corresponding author upon reasonable request.

## Supplementary Materials

The following supporting information can be downloaded at https://www.mdpi.com/article/10.3390/toxics14090760/s1, Table S1: Cell viability of MCF-7 cells exposed to 13 bioprinting materials and controls assessed by the MTT assay; Table S2: Cell viability of PALM cells exposed to 13 bioprinting materials and controls assessed by the MTT assay.

## Abbreviations

The following abbreviations are used in this manuscript:

ABS	Acrylonitrile butadiene styrene
B-TPUe	1,4-butanediol thermoplastic polyurethane elastomer
GelMA	methacrylated gelatine
HAMA	methacrylated hyaluronic acid
PCL	Polycaprolactone
PEGDA	poly(ethylene glycol) diacrylate
PLA	polylactic acid
PVA	polyvinyl alcohol
R1881	reference androgen metiltrienolone
