# Supplementary material for: Hormonal Activity of 3D Bioprinting Materials Using the E-Screen and PALM Bioassays
Source: Toxics. 2026 Aug 26;14(9):760. doi: 10.3390/toxics14090760 (PMC13611166; doi:10.3390/toxics14090760)
Supplement: Supplementary file 1 [file toxics-14-00760-s001.zip › toxics-4465341-supplementary.pdf]

Supplementary Material

Table S1. Cell viability of MCF-7 cells exposed to 13 bioprinting materials and controls assessed by the MTT assay.

Table S2. Cell viability of PALM cells exposed to 13 bioprinting materials and controls assessed by the MTT assay.

Table S1. Cell viability of MCF-7 cells exposed to 13 bioprinting materials and controls assessed by the MTT assay.

		PLA	ABS	PCL	b-TPUe	PEDGA	Hyaluronic acid	Agarose	Silk fibroin	GelMa	HAMA	PVA	Collagen	Alginate
**Estrogenic activity**	**C+**	99.85 ±0.02	99.40 ±0.03	99.10 ±0.01	100 ±0.02	99.65 ±0.01	99.20 ± 0.03	99.90 ±0.02	99.50 ± 0.01	99.75 ±0.02	99.30 ±0.03	99.80 ±0.01	100 ±0.02	99.60 ±0.01
	**C-**	100± 0.01	99.90 ±0.02	99.80 ±0.01	99.95 ±0.02	100±0.01	99.85 ± 0.02	100± 0.01	99.95 ± 0.03	100 ± 0.02	99.80 ±0.01	99.90 ±0.02	100± 0.01	99.85 ±0.02
	**10^{-9} M**						N.T.					97.45 ±0.02	98.12 ± 0.03	96.80 ±0.01
	**$3x10^{-9}$ M**						N.T.					98.30 ±0.01	97.65 ± 0.02	99.10 ±0.03
	**10^{-8} M**	97.80 ±0.02	96.50 ±0.01	98.90 ±0.03	99.15 ±0.02	95.80 ±0.01	98.40 ± 0.02	97.10 ±0.03	99.30 ± 0.01	96.90 ±0.02	98.75 ±0.01	97.20 ±0.03	96.40 ± 0.02	98.50 ±0.01
	**$3x10^{-8}$ M**	98.20 ±0.01	97.10 ±0.03	96.80 ±0.02	98.40 ±0.01	97.90 ±0.03	96.70 ± 0.01	98.85 ±0.02	97.60 ± 0.03	98.10 ±0.01	96.30 ±0.02	98.90 ±0.01	97.80 ± 0.03	97.15 ±0.02
	**10^{-7} M**	96.90 ±0.03	98.40 ±0.02	97.55 ±0.01	96.90 ±0.03	98.30 ±0.02	99.10 ± 0.01	96.40 ±0.02	98.20 ± 0.01	97.40 ±0.03	98.90 ±0.02	96.80 ±0.01	98.30 ± 0.02	96.70 ±0.03
	**$3x10^{-7}$ M**	99.10 ±0.02	96.80 ±0.01	98.30 ±0.03	97.60 ±0.02	96.40 ±0.01	97.80 ± 0.03	98.10 ±0.01	96.90 ± 0.02	98.60 ±0.03	97.50 ±0.01	98.10 ±0.02	99.00 ± 0.01	98.40 ±0.02
	**10^{-6} M**	95.70 ±0.01	98.90 ±0.03	96.20 ±0.02	98.80 ±0.01	97.10 ±0.02	96.30 ± 0.03	97.90 ±0.01	98.50 ± 0.02	96.80 ±0.01	98.20 ±0.03	97.60 ±0.02	96.90 ± 0.01	97.80 ±0.03
	**$3x10^{-6}$ M**	98.40 ±0.03	97.30 ±0.02	98.70 ±0.01	96.50 ±0.03	98.60 ±0.01	98.20 ± 0.02	96.70 ±0.03	97.10 ± 0.01	97.90 ±0.02	96.60 ±0.02		N.T.	
	**10^{-5} M**	97.20 ±0.02	96.90 ±0.01	97.80 ±0.03	97.10 ±0.02	96.90 ±0.03	97.50 ± 0.01	98.30 ±0.02	96.40 ± 0.03	98.20 ±0.01	97.80 ±0.03		N.T.	
**Anti-estrogenic activity+**	**C+**	99.70 ±0.02	99.25 ±0.03	99.80 ±0.01	99.10 ±0.02	99.90 ±0.01	99.45 ± 0.02	99.15 ±0.03	99.85 ± 0.01	99.30 ±0.02	99.60 ±0.01	99.90 ±0.02	99.10 ± 0.03	99.75 ±0.01
	**C-**	100± 0.01	99.85 ±0.02	99.90 ±0.01	100± 0.03	99.80 ±0.02	99.95 ± 0.01	100± 0.02	99.90 ± 0.01	99.85 ±0.03	100 ±0.01	99.95 ±0.02	100± 0.01	99.90 ±0.02
	**10^{-9} M**						N.T.					96.30 ±0.02	98.70 ± 0.03	97.90 ±0.01
	**$3x10^{-9}$ M**						N.T.					97.80 ±0.01	96.50 ± 0.02	98.40 ±0.03
	**10^{-8} M**	98.10 ±0.02	96.80 ±0.03	97.40 ±0.01	98.90 ±0.02	96.70 ±0.03	98.20 ± 0.01	97.60 ±0.02	99.10 ± 0.03	96.50 ±0.01	98.30 ±0.02	97.90 ±0.03	96.20 ± 0.01	98.60 ±0.02
	**$3x10^{-8}$ M**	97.30 ±0.01	98.50 ±0.02	96.90 ±0.03	97.80 ±0.01	98.20 ±0.02	96.40 ± 0.03	98.70 ±0.01	97.20 ± 0.02	98.80 ±0.03	96.90 ±0.01	98.10 ±0.02	97.40 ± 0.03	96.80 ±0.01
	**10^{-7} M**	96.80 ±0.03	97.90 ±0.01	98.30 ±0.02	96.50 ±0.03	98.80 ±0.01	99.00 ± 0.02	96.90 ±0.03	98.40 ± 0.01	97.10 ±0.02	98.60 ±0.03	96.70 ±0.01	98.90 ± 0.02	97.30 ±0.01
	**$3x10^{-7}$ M**	98.60 ±0.02	96.40 ±0.03	97.80 ±0.01	98.20 ±0.02	96.10 ±0.03	97.30 ± 0.01	98.50 ±0.02	96.80 ± 0.03	98.30 ±0.01	97.10 ±0.02	98.50 ±0.03	97.60 ± 0.01	98.20 ±0.02
	**10^{-6} M**	95.90 ±0.01	98.10 ±0.02	96.50 ±0.03	98.60 ±0.01	97.40 ±0.02	96.80 ± 0.03	97.30 ±0.01	98.90 ± 0.02	96.20 ±0.03	98.40 ±0.01	97.80 ±0.02	96.60 ± 0.03	97.50 ±0.01
	**$3x10^{-6}$ M**	98.10 ±0.03	97.60 ±0.01	98.40 ±0.02	96.90 ±0.03	98.10 ±0.01	98.50 ± 0.02	96.20 ±0.03	97.50 ± 0.01	97.70 ±0.02	96.80 ±0.03		N.T.	
	**10^{-5} M**	97.50 ±0.02	96.30 ±0.03	98.10 ±0.01	97.40 ±0.02	96.60 ±0.03	97.90 ± 0.01	98.10 ±0.02	96.70 ± 0.03	98.50 ±0.01	97.30 ±0.02		N.T.	

C+: positive control (E2 $1x10^{-10}$ M); C-: negative control (culture media); N.T.: not tested; SD: Standard deviation. +For anti-estrogenic activity, MCF-7 cells were treated with the bioprinting materials and E2 (100 pM). ABS: acrylonitrile butadiene styrene; b-TPUe: 1,4-butanediol thermoplastic polyurethane elastomer; GelMA: methacrylated gelatine; HAMA: methacrylated hyaluronic acid; PCL: polycaprolactone; PEGDA: poly(ethylene glycol) diacrylate; PLA: polylactic acid; PVA: polyvinyl alcohol.

Table S2. Cell viability of PALM cells exposed to 13 bioprinting materials and controls assessed by the MTT assay.

		PLA	ABS	PCL	b-TPUe	PEDGA	Hyaluronic acid	Agarose	Silk fibroin	GelMa	HAMA	PVA	Collagen	Alginate
**Androgenic activity**	**C+**	99.70 ± 0.02	99.70 ± 0.01	99.60 ± 0.03	99.80 ± 0.01	99.60 ± 0.01	99.80 ± 0.02	99.60 ± 0.03	99.70 ± 0.01	99.90 ± 0.02	99.60 ± 0.03	99.80 ± 0.01	99.70 ± 0.02	99.70 ± 0.01
	**C-**	99.80± 0.01	99.80 ± 0.02	99.70 ± 0.01	99.70± 0.02	99.85 ± 0.01	99.60± 0.03	99.90 ± 0.01	99.80 ± 0.02	99.80± 0.01	99.70 ± 0.02	99.85 ± 0.01	99.60± 0.02	99.80 ± 0.01
	**10^{-9} M**	N.T.										96.70 ± 0.02	97.40 ± 0.03	98.20 ± 0.01
	**$3x10^{-9}$ M**	N.T.										97.90 ± 0.03	98.60 ± 0.01	96.70 ± 0.02
	**10^{-8} M**	98.40 ± 0.01	96.20 ± 0.03	97.80 ± 0.02	99.00 ± 0.01	96.50 ± 0.03	98.10 ± 0.02	97.50 ± 0.01	98.90 ± 0.02	96.30 ± 0.03	98.10 ± 0.01	97.70 ± 0.02	96.80 ± 0.03	98.20 ± 0.01
	**$3x10^{-8}$ M**	97.10 ± 0.03	98.30 ± 0.02	96.40 ± 0.01	97.90 ± 0.03	98.60 ± 0.01	97.20 ± 0.03	98.40 ± 0.02	96.80 ± 0.01	98.50 ± 0.02	96.90 ± 0.03	98.30 ± 0.01	97.10 ± 0.02	97.60 ± 0.03
	**10^{-7} M**	98.90 ± 0.02	97.50 ± 0.01	98.10 ± 0.03	96.80 ± 0.02	97.40 ± 0.03	98.80 ± 0.01	96.70 ± 0.02	98.50 ± 0.03	97.20 ± 0.01	98.40 ± 0.02	96.50 ± 0.03	98.60 ± 0.01	96.90 ± 0.02
	**$3x10^{-7}$ M**	96.50 ± 0.01	98.10 ± 0.03	97.30 ± 0.02	98.50 ± 0.01	96.90 ± 0.02	97.60 ± 0.03	98.90 ± 0.01	97.10 ± 0.02	98.00 ± 0.03	97.80 ± 0.01	98.80 ± 0.02	97.30 ± 0.03	98.10 ± 0.01
	**10^{-6} M**	98.20 ± 0.03	96.70 ± 0.02	98.80 ± 0.01	97.10 ± 0.03	98.20 ± 0.01	96.50 ± 0.02	97.60 ± 0.03	98.30 ± 0.01	96.70 ± 0.02	98.60 ± 0.03	97.10 ± 0.01	96.40 ± 0.02	97.90 ± 0.03
	**$3x10^{-6}$ M**	97.60 ± 0.02	98.90 ± 0.01	96.70 ± 0.03	98.30 ± 0.02	97.80 ± 0.03	98.10 ± 0.01	96.30 ± 0.02	97.80 ± 0.03	97.40 ± 0.01	96.20 ± 0.02	N.T.		
	**10^{-5} M**	96.80 ± 0.01	97.20 ± 0.03	98.30 ± 0.02	96.60 ± 0.01	97.10 ± 0.02	97.90 ± 0.03	98.50 ± 0.01	96.10 ± 0.02	98.70 ± 0.03	97.50 ± 0.01	N.T.		
**Anti-androgenic activity[+]**	**C+**	99.80 ± 0.01	99.70 ± 0.02	99.60 ± 0.03	99.70 ± 0.02	99.70 ± 0.02	99.60 ± 0.03	99.80 ± 0.01	99.70 ± 0.02	99.60 ± 0.01	99.60 ± 0.03	99.60 ± 0.02	99.70 ± 0.01	99.70 ± 0.02
	**C-**	99.70± 0.02	99.80 ± 0.01	99.80± 0.01	99.80 ± 0.01	99.60± 0.01	99.70 ± 0.02	99.70 ± 0.01	99.60± 0.03	99.80 ± 0.01	99.80± 0.01	99.60 ± 0.03	99.80± 0.01	99.80 ± 0.01
	**10^{-9} M**	N.T.										97.10 ± 0.03	98.50 ± 0.01	96.70 ± 0.02
	**$3x10^{-9}$ M**	N.T.										98.50 ± 0.02	97.20 ± 0.03	98.90 ± 0.01
	**10^{-8} M**	97.60 ± 0.03	98.10 ± 0.01	96.90 ± 0.02	98.40 ± 0.03	97.20 ± 0.01	98.60 ± 0.03	97.10 ± 0.02	98.80 ± 0.01	96.80 ± 0.02	98.50 ± 0.03	97.40 ± 0.01	96.60 ± 0.02	98.10 ± 0.03
	**$3x10^{-8}$ M**	98.80 ± 0.02	96.70 ± 0.03	98.20 ± 0.01	96.90 ± 0.02	98.50 ± 0.03	96.80 ± 0.01	98.20 ± 0.03	97.50 ± 0.02	98.10 ± 0.01	97.30 ± 0.02	98.60 ± 0.03	97.90 ± 0.01	96.40 ± 0.02
	**10^{-7} M**	96.30 ± 0.01	98.60 ± 0.02	97.50 ± 0.03	98.10 ± 0.01	96.80 ± 0.02	98.30 ± 0.03	97.40 ± 0.01	98.00 ± 0.02	96.40 ± 0.03	98.80 ± 0.01	97.20 ± 0.02	98.40 ± 0.03	97.80 ± 0.01
	**$3x10^{-7}$ M**	98.10 ± 0.03	97.20 ± 0.01	98.60 ± 0.02	97.50 ± 0.03	98.30 ± 0.01	97.10 ± 0.02	98.80 ± 0.03	96.50 ± 0.01	98.70 ± 0.02	96.60 ± 0.03	98.10 ± 0.01	97.20 ± 0.02	98.50 ± 0.03
	**10^{-6} M**	97.40 ± 0.02	98.30 ± 0.03	96.30 ± 0.01	98.90 ± 0.02	97.10 ± 0.03	97.70 ± 0.01	96.80 ± 0.02	98.40 ± 0.03	97.90 ± 0.01	98.10 ± 0.02	96.90 ± 0.03	96.80 ± 0.01	97.20 ± 0.02
	**$3x10^{-6}$ M**	96.90 ± 0.01	97.40 ± 0.02	98.10 ± 0.03	96.30 ± 0.01	98.70 ± 0.02	98.00 ± 0.03	97.50 ± 0.01	97.10 ± 0.02	96.60 ± 0.03	97.90 ± 0.01	N.T.		
	**10^{-5} M**	98.30 ± 0.03	96.80 ± 0.01	97.70 ± 0.02	98.00 ± 0.03	96.20 ± 0.01	97.40 ± 0.02	98.30 ± 0.03	96.90 ± 0.01	98.10 ± 0.02	96.70 ± 0.03	N.T.		

C+: positive control (R1881 $1x10^{-7}$ M); C-: negative control (culture media); N.T.: not tested; SD: Standard deviation. + For anti-androgenic activity, PALM cells were treated with the bioprinting materials and R1881 (0.3 nM). ABS : acrylonitrile butadiene styrene ; b-TPUe : 1,4-butanediol thermoplastic polyurethane elastomer; GelMA: methacrylated gelatine; HAMA: methacrylated hyaluronic acid; PCL: polycaprolactone; PEGDA: poly(ethylene glycol) diacrylate; PLA: polylactic acid ; PVA: polyvinyl alcohol.
